# Coronal offset of C7 is associated with uneven joint degeneration between right and left hips after spinal fusion

**DOI:** 10.1038/s41598-025-10806-4

**Published:** 2025-07-11

**Authors:** Toshiyuki Kawai, Takayoshi Shimizu, Yaichiro Okuzu, Yutaka Kuroda, Yugo Morita, Bungo Otsuki, Koji Goto, Shunsuke Fujibayashi, Shuichi Matsuda

**Affiliations:** 1 Department of Orthopaedic Surgery, Graduate school of medicine, Kyoto University, 54 Kawahara-cho, Shogoin, Sakyo-ku, Kyoto City, 606-8507 Japan; 2 Department of Orthopaedic Surgery, Kindai University, Higashi-osaka, Japan

**Keywords:** Joint space narrowing, Hip arthritis, Spinopelvic parameters, Spinal fusion, C7–central sacral vertical line, Coronal offset, Diseases, Rheumatology

## Abstract

The aim of the study was to investigate the effects of coronal offset of C7 after spinal fusion on the discrepancy in joint space narrowing between the right and left hips. We retrospectively reviewed data from patients who underwent lumbar spinal fusion from 2011 to 2018 at our institute. The rate of hip joint space narrowing after spinal fusion was measured in 190 patients (380 hips). We assessed the effects of the distance between the C7 plumb line and the central sacral vertical line (C7-CSVL) on the discrepancy in joint space narrowing between the right and left hips. Using multivariate regression models, we controlled for the effects of age, sex, body mass index, fusion length, and several spinopelvic alignment parameters (sacral slope, pelvic incidence, lumbar lordosis, pelvic incidence minus lumbar lordosis, and sagittal vertical axis) on the joint space narrowing rate. Multivariate regression showed that the C7-CSVL was associated with the discrepancy of the joint space narrowing rate between right and left, indicating that when C7 deviated to right, the joint space narrowing was larger in the right than left hip (standardized coefficient, 0.203; *p* = 0.0005). A larger C7-CSVL was associated with a larger right–left discrepancy in hip joint space narrowing after spinal fusion. These findings indicate that spinal coronal balance affects the distribution of joint degeneration in the right and left hips. Surgeons should understand the potential risk of uneven progression of degeneration between the right and left hips in patients with large coronal offset.

## Introduction

Spinal fusion surgery is an established procedure that alleviates the symptoms caused by spinal problems when performed for appropriately selected patients. However, spinal fusion alters the spinopelvic biomechanics, and lumbar fusion sometimes causes adjacent segment degeneration. The incidence of symptomatic adjacent segment degeneration requiring additional spinal surgery is 16.5% at 5 years and 36.1% at 10 years after spinal fusion^1^.

Spinal fusion also affects the hip joint, which can be considered an adjacent joint of the lumbar spine. Previous research has shown that long spinal fusion causes increased force on the hip, leading to accelerated hip joint narrowing^2,3^.

Hip joint degeneration is affected by not only the fusion length but also spinopelvic alignment. A larger pelvic incidence (PI), sacral slope (SS), and PI minus lumbar lordosis (PI-LL) have been shown to be associated with an increased joint space narrowing rate (JSNR) after spinal fusion^4^. Other studies involving patients without spinal fusion showed that a larger posterior pelvic tilt (PT)^5,6^ and PI^7,8^ were associated with hip osteoarthritis (OA), whereas other studies showed no significant relationship between PI and hip OA^9,10^. Although several studies have examined the effects of sagittal alignment on hip degeneration, none have assessed the effects of coronal spinopelvic alignment on hip degeneration.

The literature suggests that a coronal offset of 0 ± 28 mm can be accepted as a non-pathologic state^11^ and it is biomechanically most energy-efficient to have the smallest possible sagittal and coronal offset. When the coronal balance is lost, the mechanical load is unevenly distributed to the right and left hips, which can result in an uneven degeneration process between the right and left hips.

Our primary purpose was to investigate the effect of coronal spinopelvic alignment on hip joint narrowing after spinal fusion. We separately analyzed whether the direction of imbalance affects the side in which hip degeneration is dominant.

The present study aimed to identify the effect of coronal balance on the JSNR in hips with the location of the C7 vertebra in standing radiographs as a representative parameter of coronal balance. The relationship of the location of the C7 vertebra to the discrepancy in the JSNR between the right and left hips was also examined.

### Patients and methods

This retrospective study included patients who underwent lumbar or lumbosacral spinal fusion at our institute. All patients provided informed consent, and the study protocol was approved by the institutional review board of our hospital (approval ID: R2901). This study was conducted in accordance with the principles of the Declaration of Helsinki.

From October 2011 to July 2018, 257 patients underwent lumbar or lumbosacral fusion at our institute. Fusion surgery involved the connection of two or more vertebral bodies using metal screws and rods. The patients included in the present study had also been part of a larger cohort in previous studies of the association between the JSNR and the spinal fusion length^3^. We restricted our enrollment to surgeries performed up to July 2018 to ensure a sufficient length of postoperative follow-up. Because joint space narrowing is expected to progress only minimally over relatively short intervals, including patients with less follow-up might have introduced substantial measurement error and obscured these subtle changes. Concluding recruitment in 2018 thus allowed us to more reliably detect small differences in joint space narrowing.

**Fig. 1:**
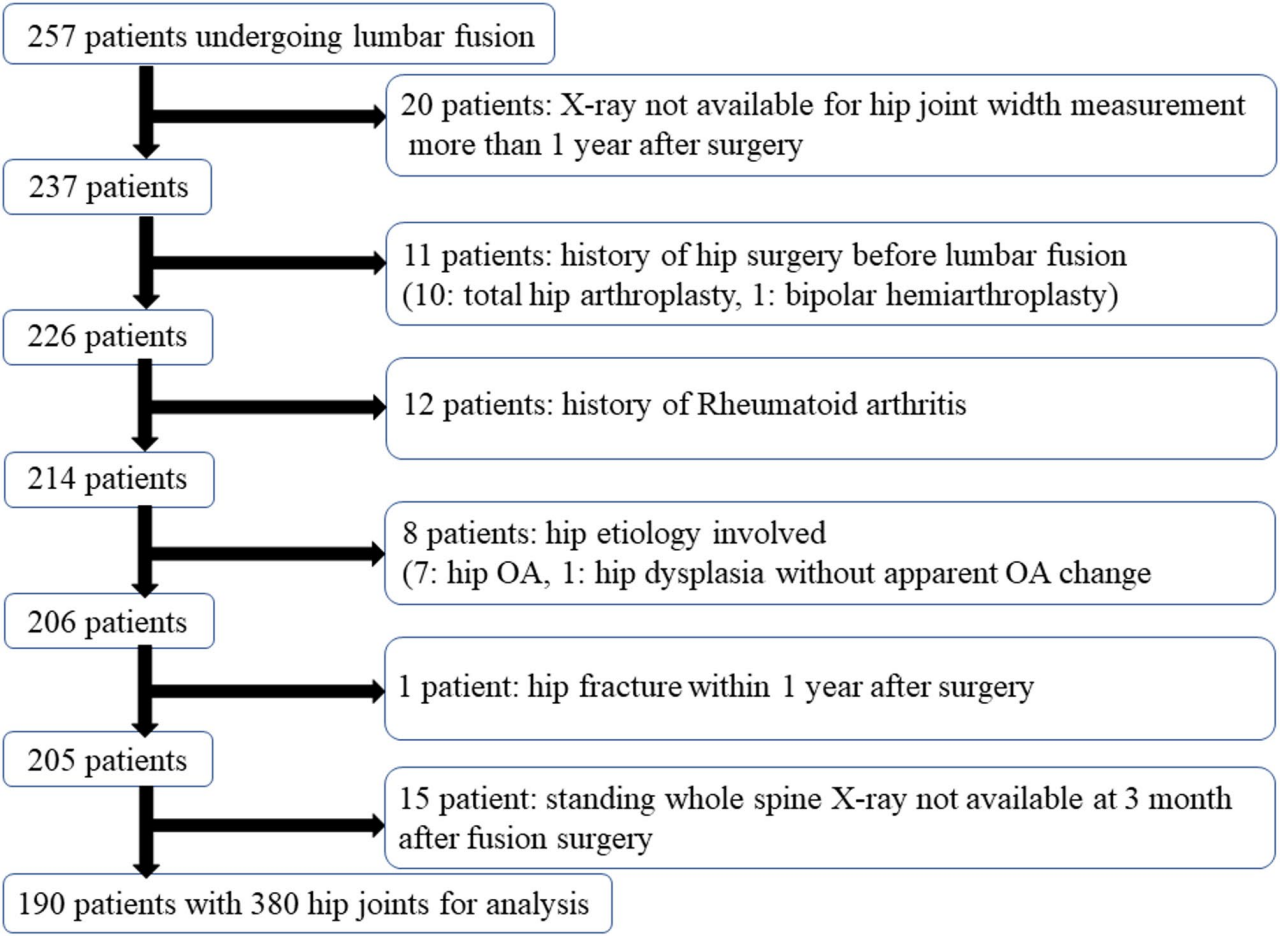
Flow chart showing patient inclusion and exclusion. OA, osteoarthritis.

The inclusion criterion was having undergone lumbar spinal fusion at our institution during the study period. Patients were excluded if they did not have available standing radiographs (including measurable hip joints) taken more than one year after surgery, had a history of hip surgery, had rheumatoid arthritis, had hip osteoarthritis (Kellgren–Lawrence grade ≥ II), had hip dysplasia (defined as a center–edge angle < 20°), sustained a hip fracture within one year following spinal surgery, or did not undergo postoperative whole-spine standing radiography at three months after the fusion procedure. Of the 257 patients, 67 were excluded because they lacked available standing radiographs at more than 1 year after spinal surgery that included measurable hip joints, had a history of hip surgery, had rheumatoid arthritis, had hip OA (Kellgren–Lawrence grade ≥ II^12^), had hip dysplasia (center–edge angle of < 20°), sustained a hip fracture within 1 year after spinal surgery, or lacked available postoperative whole-spine standing radiographs at 3 months after fusion surgery (Fig. 1). The final cohort comprised 190 patients (380 hips) in whom the joint space width of both hips was measurable at 3 months and at > 1 year postoperatively on standing whole-spine radiographs.

The indications for spinal fusion were degenerative scoliosis (*n* = 38), degenerative disc disease (*n* = 124), spondylolisthesis (*n* = 24), and osteoporotic vertebral collapse (*n* = 4).

Radiographs were taken in a previously described manner^4^. Briefly, anteroposterior and lateral standing radiographs of the whole spine (including the pelvis), standardized for the beam position and radiographic penetration, were obtained preoperatively; at 3, 6, and 12 months postoperatively; and annually thereafter. Lateral radiographs were taken in the standing position with the hands supported while flexing the shoulders 30 degrees.

### Measurements of spinopelvic parameters

**Fig. 2:**
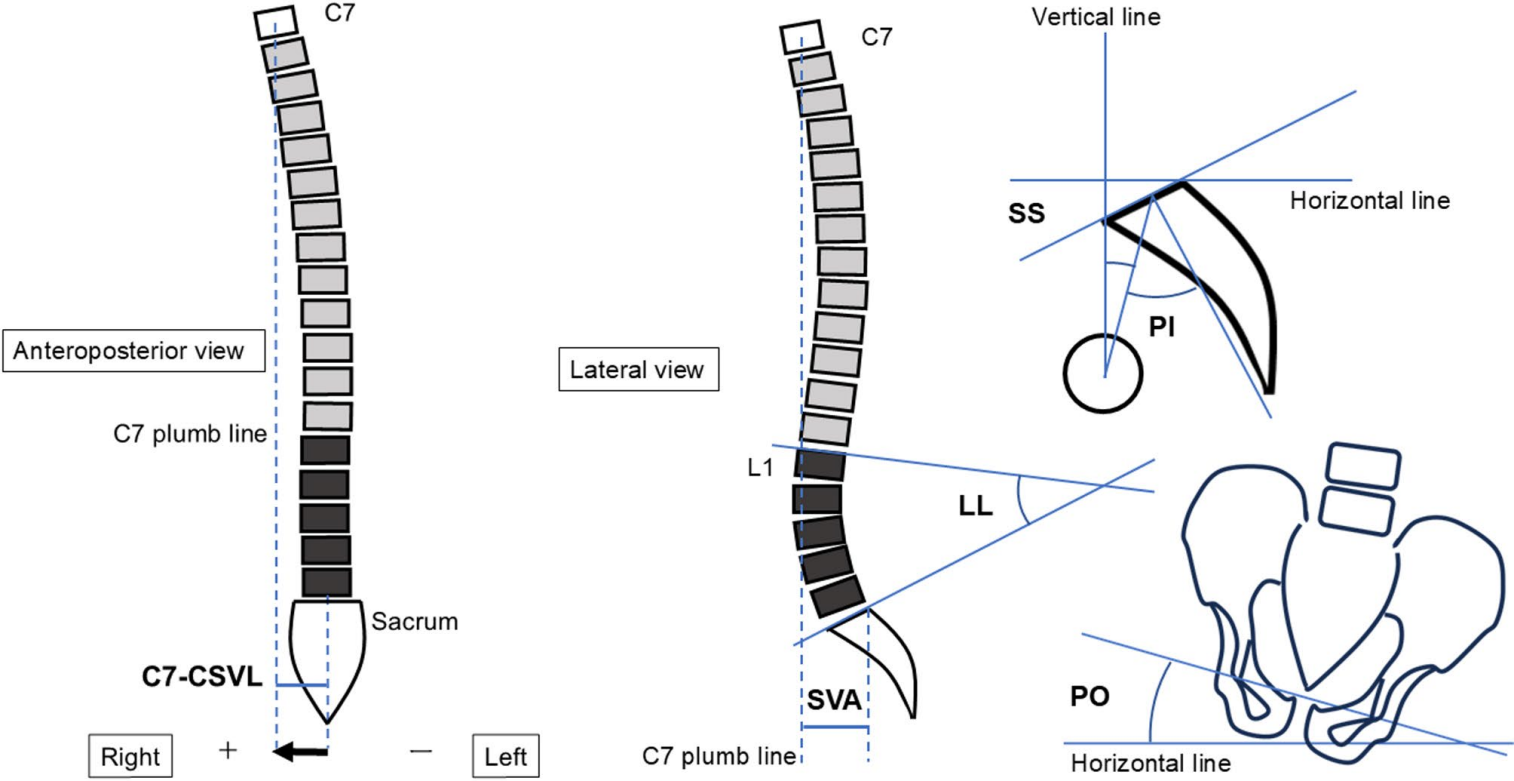
Shema showing the measurements of PT, PI, SS, LL, SVA, PO and C7-CSVL. PI was defined as the angle between the line perpendicular to the sacral plate at its midpoint and the line connecting this point to the center of the axis of the femoral heads. SS was defined as the angle between a line tangential to the upper S1 endplate and a horizontal line. LL was defined as the angle between the line of the L1 superior endplate and the line of the S1 superior endplate. The SVA was defined as the horizontal distance between a vertical line drawn from the center of the C7 vertebral body and the posterosuperior corner of S1. The PO angle was defined as the angle between the inter-teardrop line and the horizontal reference line. The absolute C7-CSVL was defined as the absolute value of C7-CSVL. C7-CSVL was the distance between the C7 plumb line and the central sacrum vertical line. Relative C7-CSVL was expressed as a positive value when the C7 plumb line passed to the right of the CSVL and as a negative value when it passed to the left. LL, lumbar lordosis; PI, pelvic incidence; SS, sacral slope; SVA, sagittal vertical axis; PO, pelvic obliquity; CSVL, central sacral vertical line.

Spinopelvic parameters were measured using standing whole-spine radiographs at 3 months after surgery. The measurements were performed using Web-based Centricity software (GE Healthcare, Chicago, IL). LL, PT, PI, SS, and C7 sagittal vertical axis (SVA) were evaluated as previously described (Fig. 2)^13^. The PI-LL mismatch (PI-LL) was additionally calculated using the difference between PI and LL. The reliability of these measurement methods was demonstrated in our previous study. The intraclass correlation coefficients for the inter- and intra-reader reliabilities of each spinopelvic parameter measurement were > 0.9^4^.

The distance between the C7 plumb line and the central sacral vertical line (C7-CSVL) was measured on anteroposterior standing radiographs (Fig. 2). The relative C7-CSVL was expressed as a positive value when the C7 plum line coursed to the right of the CSVL and as a negative value when the plum line coursed to the left the CSVL. The absolute C7-CSVL was defined as the absolute value of the relative C7-CSVL and was always expressed as a positive value regardless of which side of the CSVL the C7 plum line was located. Pelvic obliquity (PO) was assessed on standardized standing anteroposterior (AP) pelvic radiographs taken with the patient in a neutral upright position. The PO angle was defined as the angle between the inter-teardrop line and the horizontal reference line. This method has demonstrated high reliability in adult populations^14^. Pelvic obliquity (PO) was defined as positive when the right iliac crest was positioned more cranially, and negative when the left iliac crest was more cranially elevated.

### Measurement of JSNR

**Fig. 3:**
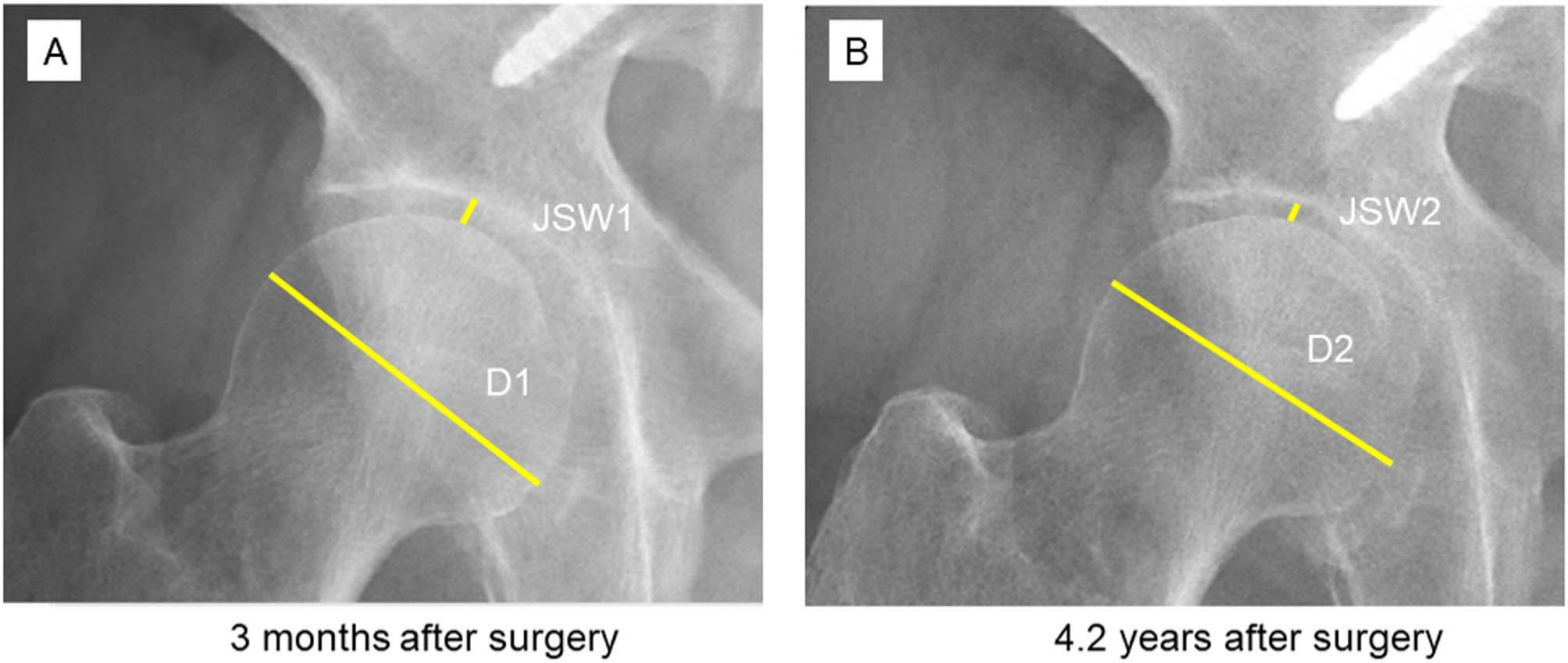
The joint space width and femoral head diameter were measured on anteroposterior standing whole-spine radiographs as indicated by the yellow lines. JSW1 and JSW2 are the joint space widths at 3 months after surgery and at the final follow-up, respectively, and D1 and D2 are the femoral head diameters measured on radiographs at 3 months after surgery and at the final follow-up, respectively.

Joint narrowing was measured on standing radiographs as described previously^3^. Briefly, the joint space width was defined as the narrowest point between the cortical surface of the acetabulum and the bone contour of the femoral head on a digitized image created using Web-based Centricity software (GE Healthcare) and magnified to fit the display (Fig. 3). The change in the joint space width was calculated as the decrease in the ratio of the joint space width to the size of the femoral head to calibrate the magnification of the radiograph and normalize the effect of body size. The change in the ratio was divided by the follow-up period to calculate the normalized JSNR (nJSNR) as shown below.

$$\:nJSNR=\frac{\left(\frac{JSW1}{D1}\:-\:\frac{JSW2}{D2}\right)}{follow-up\:duration\:in\:years}\:\times\:\:1000$$

where JSW1 and JSW2 are the joint space widths at 3 months after surgery and at the final follow-up, respectively, and D1 and D2 are the diameters of the femoral head measured on radiographs obtained at 3 months after surgery and at the final follow-up, respectively (Fig. 3).

Next, the discrepancy in the nJSNR between the right and left hips (R-L discrepancy in nJSNR) was calculated as follows:

$$\:R-L\:discrepancy\:in\:nJSNR=nJSNR\:for\:right\:hip-nJSNR\:for\:left\:hip\:$$

Both the relative and absolute values of R-L discrepancy in nJSNR were obtained and used for analysis. R-L discrepancy in nJSNR was positive when nJSNR was larger in the right than left hip.

The ratio of the joint space width to the diameter of the femoral head was defined as JSW/D. The discrepancy in JSW/D between the right and left hips was also calculated.

Measurements were performed in random order by an experienced orthopedic surgeon (T.K.). The reliability of the measurement method was demonstrated in our previous study. The intraclass correlation coefficients for inter- and intra-reader reliabilities of the joint space measurements were 0.91 and 0.93, respectively^4^.

### Statistical analysis

For comparison of demographic data, the patients were divided into two groups: the balanced group (C7-CSVL of < 30 mm) and imbalanced group (C7-CSVL of < 30 mm) based on a previous study of coronal balance^11^. The effects of each parameter on R-L discrepancy in nJSNR were analyzed for all patients.

Differences in proportions were calculated using the Pearson chi-square test. Differences in means were calculated using the Wilcoxon test for comparison of two groups or the Kruskal–Wallis test followed by the post-hoc Steel–Dwass test for comparison of more than two groups. Univariate and multivariate regression analyses were performed to determine the association between each of the following factors and the relative or absolute value of R-L discrepancy in nJSNR: age, sex, body mass index (BMI), number of fusion levels, PI, PT, LL, PI-LL, SS, SVA, and relative or absolute C7-CSVL. Interactions were quantified using variance inflation factors (VIFs), with values of 5 to 10 indicating collinearity. Statistical significance was set at 0.05. PT and LL were excluded from the multivariate models because of probable strong correlations with other variables (based on an unacceptably high VIF obtained in test examinations). PT is theoretically correlated with SS, and surgeons tend to determine the target LL according to the PI. Both PI and PI-LL were included in the multivariate models because an acceptably low VIF was obtained.

Because no previous studies have investigated this correlation, it was difficult to predict the anticipated correlation coefficient. Therefore, we assumed a correlation coefficient of 0.25. Based on this assumption, with a significance level (α) of 0.05 and a power of 80%, the required sample size was estimated to be approximately 124. However, taking into account potential confounders—such as the number of fused vertebrae—we elected to recruit 190 patients to ensure a sufficient sample size.

All statistical analyses were performed using JMP Pro 15 software (URL: https://www.jmp.com/en/software) (SAS Institute, Cary, NC).

## Results

The patients’ demographic data are shown in Table 1. The mean follow-up duration was 4.1 ± 1.7 years. Postoperative C7-CSVL was < 30 mm in 170 patients (balanced group) and > 30 mm in the remaining 20 patients (imbalanced group). The patients in the balanced group tended to be younger (69.5 vs. 71.0 years, *p* = 0.059), had undergone significantly shorter fusions (2.85 vs. 4.35 fusion levels, *p* = 0.019), and had a smaller nJSNR (2.283 vs. 3.861, *p* < 0.001) and a smaller absolute discrepancy in nJSNR between the right and left hips (1.626 vs. 2.419, *p* = 0.040). There was no significant difference in sex, BMI, or indication.

**Table 1:** Patient demographics.

		All subjects (*n* = 190)	Balanced group C7CSVL < 30 mm (*n* = 170)	Imbalance group C7CSVL > 30 mm (*n* = 20)	*P* (Balanced vs. Imbalanced)
Age		70.0 ± 10.2 (34–93)	69.5 ± 9.8 (34–87)	71.0 ± 13.2 (38–93)	0.059
Sex (male)		72/190 (38.0%)	66/170 (38.8%)	6/20 (30.0%)	0.28
BMI (kg/m^2^)		24.0 ± 3.9 (19.9–28.2)	23.9 ± 3.8 (16.2 36.1)	24.6 ± 5.2 (15.7 32.8)	0.46
Indication	DDD	124	111(65.3%)	13(65.0%)	0.22
	Spondylolisthesis	24	23(13.5%)	1(5.0%)	
	Scoliosis	38	33(19.4%)	5(25.0%)	
	Osteoporotic vertebral compression fracture	4	3(1.8%)	1(5.0%)	
Number of levels of fusion		3.01 ± 2.80 (1, 13)	2.85 ± 2.61 (1, 13)	4.35 ± 3.67 (1, 12)	0.019
nJSNR		2.449 ± 3.561 (−3.251, 23.933)	2.283 ± 3.557 (−3.251, 23.933)	3.861 ± 3.317 (−0.808, 17.008)	< 0.001
Absolute value of R-L discrepancy in nJSNR		1.710 ± 1.774 (0.008, 10.270)	1.626 ± 1.655 (0.008, 8.253)	2.419 ± 2.484 (0.113, 10.270)	0.044

Values are expressed as mean ± standard deviation (minimum, maximum).

BMI indicates body mass index; DDD, degenerative disc disease; CSVL, central sacral vertical line; nJSNR, normalized joint space narrowing rate.

**Fig. 4:**
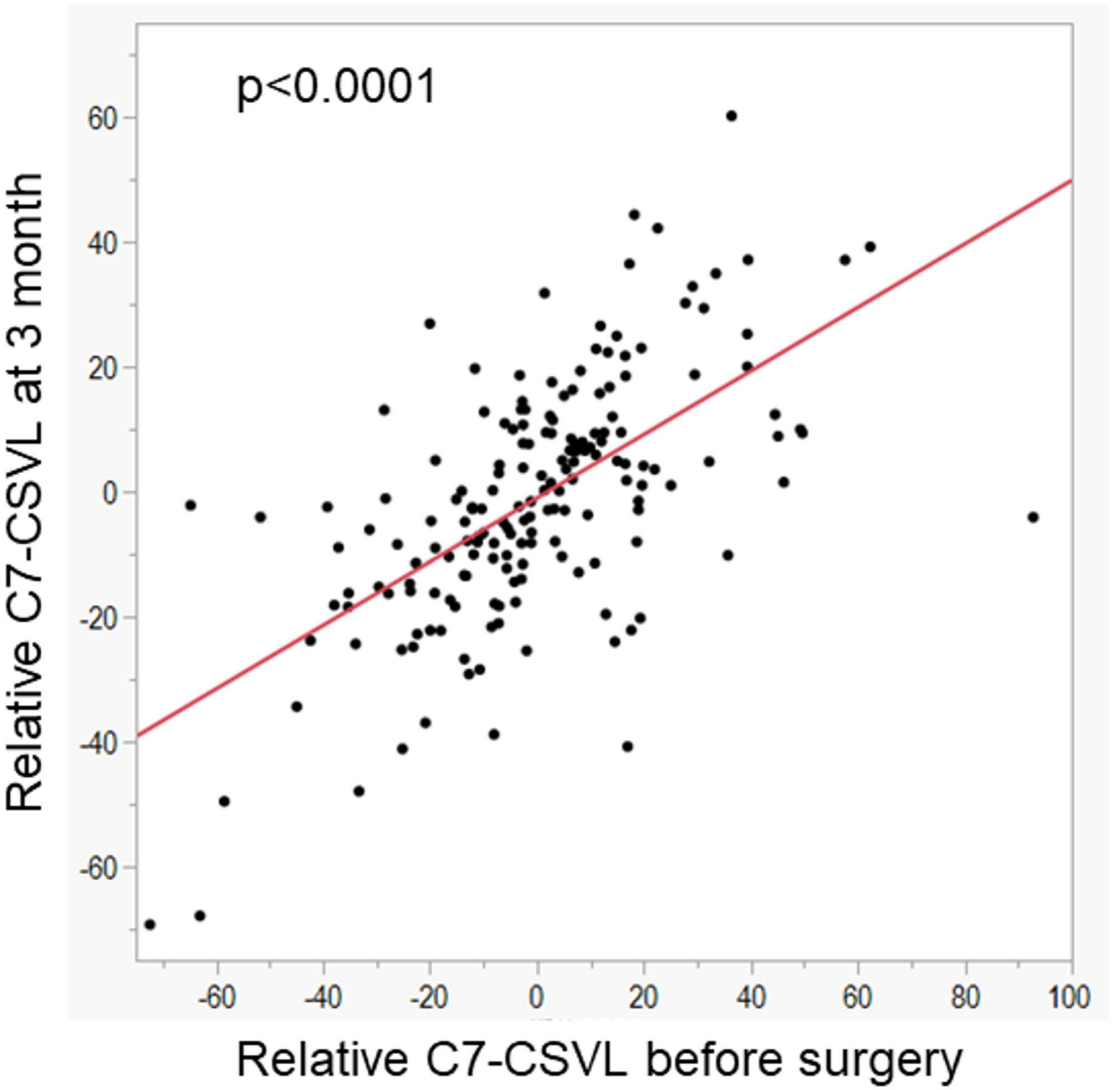
Relationship between relative value of C7-CSVL before surgery and relative C7-CSVL at 3 months. CSVL, central sacral vertical line.

The relative value of C7-CSVL at 3 months was significantly correlated with the preoperative relative value of C7-CSVL (*p* < 0.0001) (Fig. 4). The absolute changes in relative C7CSVL from 3 months postoperatively to 1 year and to the final follow-up were 7.4 ± 8.0 mm and 11.6 ± 15.1 mm, respectively (mean ± SD). Relative C7-CSVL values at 3 months, 1 year, and the final follow-up were significantly correlated with each other, with all pairwise correlations demonstrating strong associations (Spearman’s ρ > 0.7 for all pairs; Table 2).

**Table 2:** Correlation analysis of relative C7-CSVL across all combinations of time points.

	*P* value		ρ	
	3 months	1 year	3 months	1 year
3 months				
1 year	< 0.0001		0.722	
Last follow-up	< 0.0001	< 0.0001	0.712	0.767

CSVL, central sacral vertical line.

Pelvic obliquity (PO) was not correlated with the relative value of R–L discrepancy in nJSNR (*p* = 0.493, ρ = 0.050). Therefore, in the following analyses, C7-CSVL was used as the representative parameter for coronal balance.

Univariate regression performed using the absolute value of C7-CSVL as the dependent variable showed that a higher PI-LL, a larger number of fusion levels, and a higher postoperative SVA were associated with a larger absolute value of C7-CSVL, while sex, age, BMI, postoperative SS, and postoperative PI were not (Table 3).

**Table 3:** Results of univariate regression analyses performed using the rate of absolute value of C7-CSVL as the dependent variable (for 190 patients, 380 hips).

	Univariate testing			
	t	Standard error	Standardized coefficients beta	*p*-value
Sex (male)	−0.69	1.34	−0.0353	0.49
Age	1.15	0.0639	0.0589	0.25
BMI	1.63	0.194	0.0930	0.10
**Number of fusion levels**	2.58	0.233	0.131	**0.010**
PI	1.74	0.0661	0.0893	0.082
**PT**	3.31	0.0605	0.168	**0.0010**
LL	−3.23	0.0458	−0.164	0.0014
**PI-LL**	4.20	0.0423	0.211	**< 0.0001**
SS	−1.73	0.0637	−0.0887	0.084
**SVA**	4.02	0.0118	0.202	**< 0.0001**
**Absolute value of PO**	2.60	0.7444	0.186	**0.010**

CSVL, central sacral vertical line; BMI indicates body mass index; PI, pelvic incidence; PT, pelvic tilt; LL, lumbar lordosis; SS, sacral slope; SVA, sagittal vertical axis; PO, pelvic obliquity.

Univariate regression analyses performed with the absolute value of R-L discrepancy in nJSNR as the dependent variable demonstrated that female sex (*p* = 0.0011), a larger number of fusion levels (*p* < 0.0001), larger PI (*p* = 0.011), and higher absolute value of C7-CSVL (*p* = 0.0083) were associated with a higher absolute value of R-L discrepancy in nJSNR (Table 4). In the multivariate model, a larger number of fusion levels [*p* < 0.0001; standardized coefficient (SC) = 0.254], larger PI (*p* = 0.025, SC = 0.201), and larger absolute value of C7-CSVL (*p* = 0.012, SC = 0.140) were the independent factors associated with an increased absolute value of R-L discrepancy in nJSNR.

**Table 4:** Results of univariate and multivariate regression analyses performed using the rate of absolute value of R-L discrepancy in nJSNR as the dependent variable (for 190 patients, 380 hips).

	Univariate testing				Multivariate model				VIF
	t	Standard error	Standardized coefficients beta	*p*-value	t	Standard error	Standardized coefficients beta	*p*-value	
Sex (male)	−3.28	0.185	−0167	**0.0011**	−1.85	0.219	−0.106	0.066	1.173
Age	1.03	0.00896	0.0530	0.30	−0.20	0.0102	0.0118	0.84	1.258
BMI	−1.69	0.0265	−0.0967	0.091	−1.82	0.0279	−0.109	0.070	1.285
Number of fusion levels	5.22	0.0318	0.259	**< 0.0001**	4.60	0.0344	0.254	**< 0.0001**	1.092
PI	2.57	0.00922	0.131	**0.011**	2.25	0.0159	0.201	**0.025**	2.828
PT	1.16	0.00860	0.0596	0.25					
LL	2.52	0.00646	0.129	**0.012**					
PI-LL	−0.67	0.00607	−0.0346	0.50	−1.42	0.0107	−0.129	0.16	2.935
SS	1.25	0.00895	0.0642	0.21	−0.24	0.0141	−0.0193	0.81	2.325
SVA	−1.36	0.00169	−0.0700	0.17	−1.00	0.00231	−0.0690	0.32	1.688
absolute C7-CSVL	2.65	0.00715	0.135	**0.0083**	2.54	0.00756	0.140	**0.012**	1.081

nJSNR, normalized joint space narrowing rate; BMI indicates body mass index; PI, pelvic incidence; PT, pelvic tilt; LL, lumbar lordosis; SS, sacral slope; SVA, sagittal vertical axis; CSVL, central sacral vertical line; VIF, variance inflation factor.

**Fig. 5:**
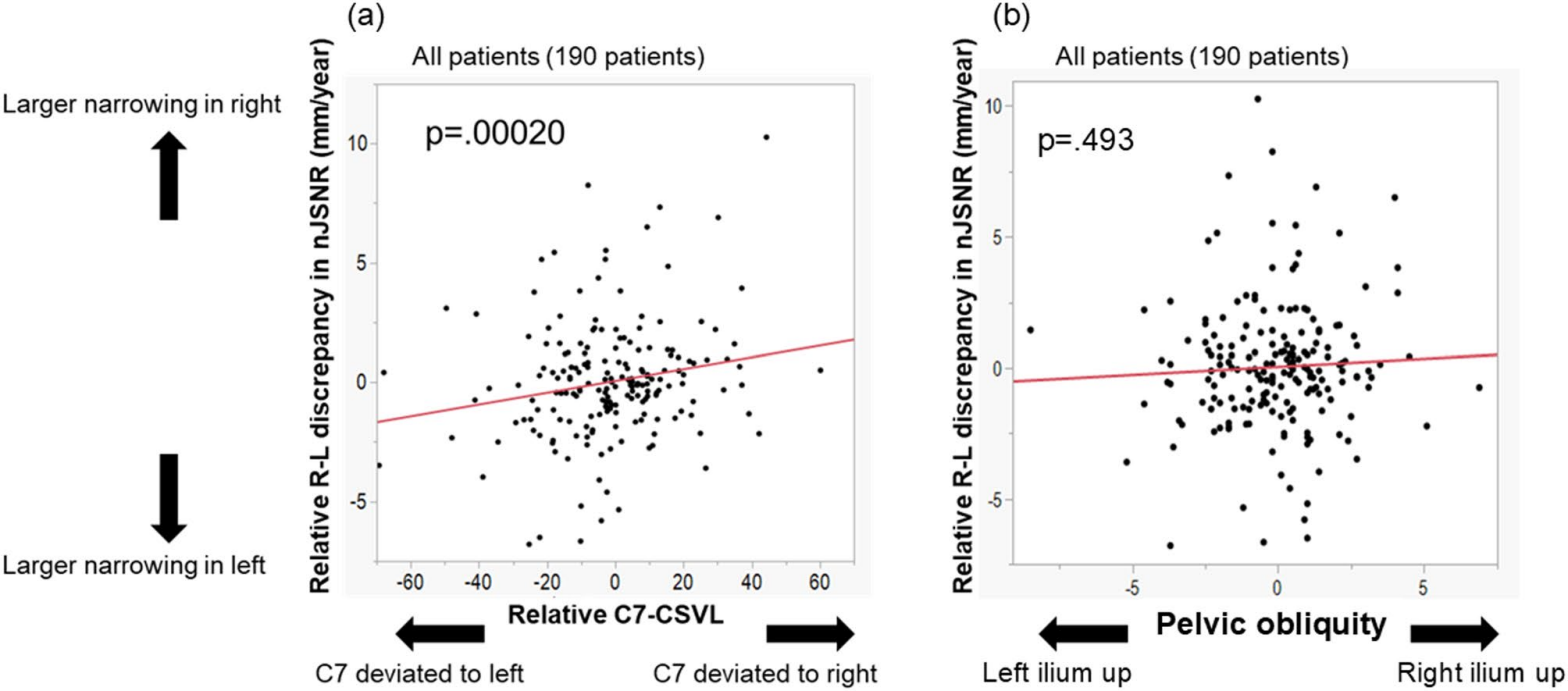
(**a**) Relationship between relative value of C7-CSVL and relative value of R-L discrepancy in nJSNR. (**b**) Relationship between the relative value of C7-CSVL and PO. CSVL, central sacral vertical line; nJSNR, normalized joint space narrowing rate; PO, pelvic obliquity.

**Fig. 6:**
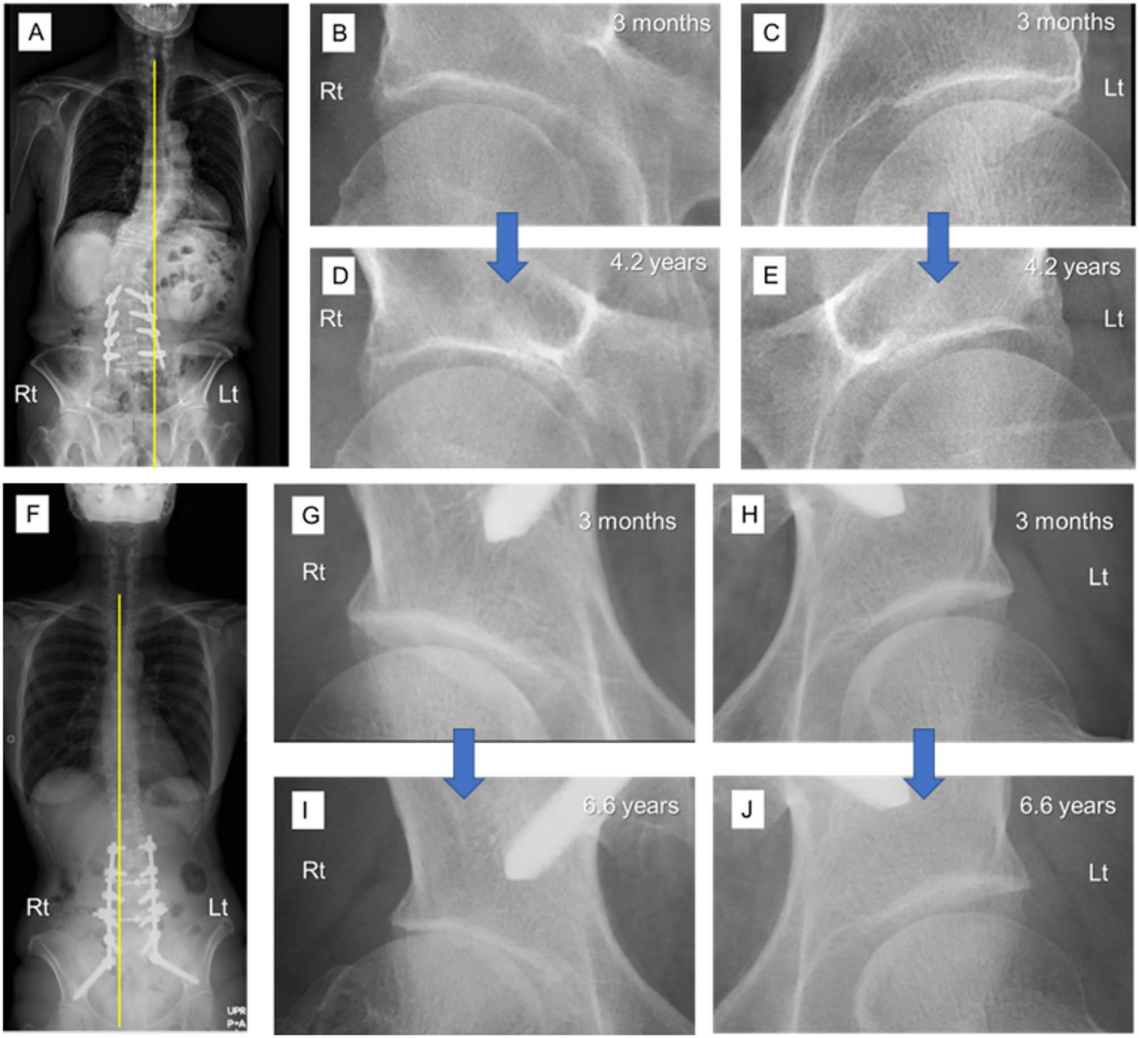
(**A**–**E**) Images of a 74-year-old man who underwent L2-5 fusion. (**A**) Anteroposterior view of standing radiograph at 3 months after surgery. Yellow line indicates the C7 plumb line. His relative C7-CSVL was − 47.1 mm. (**B**, **C**) Magnified images of hip joints at 3 months. (**D**, **E**) Magnified images of hip joints at 4.2 years after surgery. His relative R-L discrepancy in nJSNR was − 2.326. (**F**–**J**) Images of a 44-year-old woman who underwent L2–iliac fusion. (**F**) Anteroposterior view of standing radiograph at 3 months after surgery. Yellow line indicates the C7 plumb line. Her relative C7-CSVL was + 15.4 mm. (**G**, **H**) Magnified images of hip joints at 3 months. (**I**, **J**) Magnified images of hip joints at 6.6 years after surgery. Her relative R-L discrepancy in nJSNR was + 4.851.

Univariate and multivariate regression analyses performed using the relative value of R-L discrepancy in nJSNR as the dependent variable are shown in Table 5. The relative value of C7-CSVL was associated with the relative value of R-L discrepancy in nJSNR (*p* = 0.0005, SC = 0203 in the multivariate model) (Table 5), meaning that when C7 deviated to the right, the JSNR was larger on the right than left side. The relationship between the relative C7-CSVL and relative R-L discrepancy in nJSNR is depicted in Fig. 5. Representative cases are shown in Fig. 6.

**Table 5:** Results of univariate and multivariate regression analyses performed using the rate of relative value of R-L discrepancy in nJSNR as the dependent variable (for 190 patients, 380 hips).

	Univariate testing				Multivariate model				VIF
	t	Standard error	Standardized coefficients beta	*p*-value	t	Standard error	Standardized coefficients beta	*p*-value	
Sex (male)	−0.21	0.261	−0.0106	0.84	−1.15	0.316	−0.0696	0.25	1.165
Age	−1.49	0.0124	−0.0767	0.14	−0.87	0.0148	−0.0547	0.38	1.259
BMI	1.42	0.0365	0.0814	0.16	1.08	0.0405	0.0682	0.28	1.281
Number of fusion levels	2.39	0.0454	0.122	**0.017**	1.61	0.0498	0.0937	0.11	1.086
PI	1.03	0.0129	0.0531	0.30	0.20	0.0231	0.0191	0.84	2.832
PT	0.67	0.0120	0.0345	0.50					
LL	0.17	0.00905	0.00857	0.87					
PI-LL	0.52	0.00844	0.0267	0.60	0.50	0.0156	0.0482	0.62	2.939
SS	0.30	0.0125	0.0152	0.77	0.62	0.0205	0.0532	0.53	2.332
SVA	−0.19	0.00236	−0.00955	0.85	−1.15	0.00338	−0.0844	0.25	1.713
Relative C7CSVL	3.77	0.00654	0.190	**0.0002**	3.54	0.00724	0.203	**0.0005**	1.044

nJSNR, normalized joint space narrowing rate; BMI indicates body mass index; PI, pelvic incidence; PT, pelvic tilt; LL, lumbar lordosis; SS, sacral slope; SVA, sagittal vertical axis; CSVL, central sacral vertical line; VIF, variance inflation factor.

**Fig. 7:**
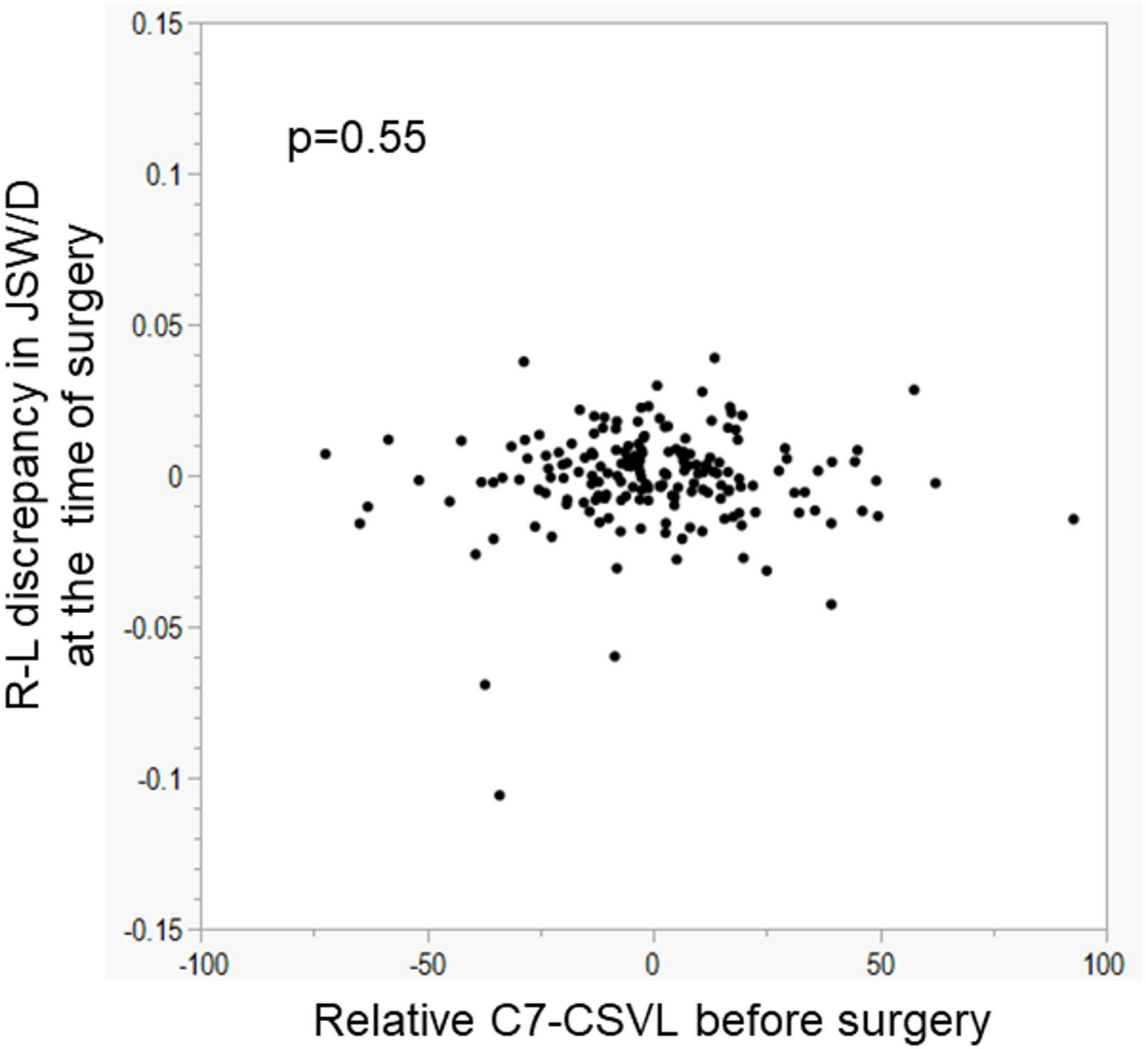
Relationship between relative value of C7-CSVL before surgery and R-L discrepancy in JSW/D at the time of the index surgery. CSVL, central sacral vertical line; JSW, joint space width; D, diameter of femoral head.

To examine the effects of the preoperative coronal offset of C7 on difference in wear rate between the right and left hips the until the time of the index surgery, the association between the preoperative C7-CSVL and the discrepancy in JSW/D between the right and left hip at the time of spine surgery was analyzed. As shown in Fig. 7, the preoperative C7-CSVL was not associated with R-L discrepancy in JSW/D (*p* = 0.55) at the time of surgery. This finding suggests that the preoperative coronal offset of C7 did not cause differences in the wear rate between the two hips the until the time of the index surgery in this cohort.

## Discussion

The present study demonstrated that a larger absolute value of C7-CSVL was associated with a greater absolute R-L discrepancy in nJSNR, even after controlling for the effects of age, fusion length, and other confounding factors. This indicates that coronal offset was associated with uneven degeneration between the right and left hips, probably because of the uneven distribution of mechanical stress on both hips.

Additionally, a larger relative value of C7-CSVL was correlated with a greater relative R-L discrepancy in nJSNR. This finding indicates that greater deviation of C7 to the right after spinal fusion was associated with greater joint space narrowing in the right than left hip.

When the patients were divided into a balanced group and imbalanced group with an absolute C7-CSVL of 30 mm as the threshold, the mean JSNR was larger in the imbalanced group (Table 1). However, it would be difficult to conclude that C7-CSVL is an independent factor that increases joint space narrowing. The absolute C7-CSVL was associated with longer fusion, a larger postoperative PI-LL, and a larger SVA. Because longer fusion, a larger PI, and a larger PI-LL are reportedly associated with an increased postoperative JSNR in the hip^3,4^, the effects of C7-CSVL should be analyzed by controlling for the effects of PI, PI-LL, and fusion length. A previous study of the effects of C7-CSVL on joint space narrowing in the hip controlled for the effects of age, sex, BMI, and fusion length and demonstrated that C7-CSVL was not associated with joint space narrowing^4^.

In the present study, the multivariate regression model performed as an absolute value of R-L discrepancy in nJSNR showed that a larger C7-CSVL was associated with a larger absolute value of R-L discrepancy in nJSNR even after controlling for the effects of patient factors, including other spinopelvic parameters (Table 4).

A larger PI and larger number of fusion levels were also associated with the absolute value of R-L discrepancy in nJSNR. Because a larger PI and number of fusion levels are known to increase the nJSNR^4^, a larger nJSNR might lead to a larger R-L discrepancy in nJSNR.

Multivariate analysis performed with the relative value of R-L discrepancy as a dependent variable demonstrated that the relative C7-CSVL was the only factor correlated with the relative R-L discrepancy (Table 5). The relative C7-CSVL was positively correlated with the relative R-L discrepancy in nJSNR. This means that when the C7 plumb line coursed to the right of the CSVL, the joint space narrowing was larger in the right than left hip. This occurred because C7 deviation causes the center of gravity of the upper body to deviate to the same side, which increases the mechanical force on the hip joint of the same side while standing.

When the center of gravity of the upper body deviates to the right, the mechanical load on the right increases while that on the left decreases. By contrast, the mechanical load increases on the left while standing on the left leg alone if the center of gravity remains on the right side throughout the walking process without a limp or the use of a supportive device; this is because more power is required for the abduction muscles when the center of gravity is located more distant from the hip joint in the single-leg support period during the gait. In this study, we could not determine whether the deviation of C7 was maintained during the gait because a dynamic gait analysis was not performed.

The preoperative C7-CSVL was not associated with R-L discrepancy in JSW/D (*p* = 0.55) at the time of surgery. This means that the right and left hips had a similar joint space width regardless of coronal offset of C7 until the time of spine surgery. It suggests that coronal offset before surgery did not cause differences in joint space narrowing between the right and left hips the until the time of the index surgery; the discrepancy between the right and left occurred after spinal fusion. However, patients with hip OA or dysplasia were excluded from this study. Therefore, we cannot generalize this finding to all patients regarding the effect of the preoperative C7-CSVL on R-L discrepancy before spine surgery. Further studies are needed to clarify the responsiveness of the hip joint cartilage to coronal offset before spinal fusion.

No reports have addressed the relationship between coronal offset and hip joint degeneration. In the present study, deviation of C7 to the right was associated with greater joint narrowing in the right than left hip. A large coronal offset might have led to an uneven distribution of mechanical stress that resulted in an uneven JSNR between the right and left hips. However, the evaluation was performed using static standing radiographs. Further study of radiographic measurements combined with dynamic gait analysis is required to elucidate the relationship between coronal balance and hip joint narrowing.

In this study, patients with Kellgren–Lawrence grade ≥ II OA in either hip were excluded so that the JSNR would not be affected by the OA stage. In one study, the JSNR for non-arthritic hips was 0.06 mm/year^3^. Other researchers who measured hip joint space narrowing in patients with hip OA obtained mean rates of 0.13 to 0.30 mm/year^15^^[,16^. Among patients who underwent total hip arthroplasty for hip OA, the preoperative JSNR was as high as 0.43 ± 0.43 mm/year^17^. These findings imply that once osteoarthritic change has appeared, the narrowing speed increases as the stage progresses.

This study had several limitations. First, this study had a small sample size and was retrospective in nature, which were unavoidable considering the study design and scope. Second, the patients had non-arthritic, non-dysplastic hips. Different findings might be obtained in patients with hip OA, hip dysplasia, or femoroacetabular impingement. Third, we did not evaluate pain, hip joint function, or daily activity; further studies utilizing hip scoring tools are warranted. Fourth, the spinal parameter used in this study was measured only once (3 months after surgery). The potential change in C7-CSVL after that time point was not taken into consideration. Finally, we did not examine which preoperative parameters might be associated with C7-CSVL; we only examined the effects of postoperative C7-CSVL on hip degeneration on the right and left.

## Conclusion

A larger relative C7-CSVL was associated with a larger R-L discrepancy in nJSNR in the hips after spinal fusion. This finding indicates that when C7 deviates to one side, the hip joint space narrowing is larger on the same side than on the contralateral side. This is the first study to show the effects of spinal coronal offset on the distribution of joint degeneration in both hip joints. Surgeons should be aware of the potential risk of uneven progression of degeneration between the right and left hips in patients whose coronal balance has been lost.

## Data Availability

The datasets used and/or analysed during the current study available from the corresponding author on reasonable request.

## References

[1] Ghiselli, G. et al. Adjacent segment degeneration in the lumbar spine. *J. Bone Joint Surg. Am.* **86**, 1497–1503 (2004). 15252099 10.2106/00004623-200407000-00020

[2] Lum, Z. C. et al. Female sex and longer fusion constructs significantly increase the risk of total hip arthroplasty following spinal fusion. *J. Bone Joint Surg. Am.* **101**, 675–681 (2019). 30994584 10.2106/JBJS.18.00667

[3] Kawai, T. et al. Number of levels of spinal fusion associated with the rate of Joint-Space narrowing in the hip. *J. Bone Joint Surg. Am.* **103**, 953–960 (2021). 33770019 10.2106/JBJS.20.01578

[4] Kawai, T. et al. The impact of spinopelvic parameters on hip degeneration after spinal fusion. *Spine (Phila Pa. 1976)*. **47**, 1093–1102 (2022). 35125459 10.1097/BRS.0000000000004340

[5] Yasuda, T. et al. Characterization of rapidly progressive osteoarthritis of the hip in its early stage. *Eur. J. Rheumatol.* **7**, 130–134 (2020). 32384049 10.5152/eurjrheum.2020.19159 PMC7431352

[6] Morimoto, T. et al. Sagittal spino-pelvic alignment in rapidly destructive coxarthrosis. *Eur. Spine J. Off Publ Eur. Spine Soc. Eur. Spinal Deform Soc. Eur. Sect. Cerv. Spine Res. Soc.* **27**, 475–481 (2018). 10.1007/s00586-017-5282-5 28840349

[7] Gebhart, J. J. et al. Relationship between pelvic incidence and osteoarthritis of the hip. *Bone Joint Res.* **5**, 66–72 (2016). 26912384 10.1302/2046-3758.52.2000552 PMC4852787

[8] Yoshimoto, H. et al. Spinopelvic alignment in patients with osteoarthrosis of the hip: a radiographic comparison to patients with low back pain. *Spine (Phila Pa. 1976)*. **30**, 1650–1657 (2005). 16025036 10.1097/01.brs.0000169446.69758.fa

[9] Raphael, I. J. et al. Pelvic incidence in patients with hip osteoarthritis. *Arch. Bone Jt. Surg.* **4**, 132–136 (2016). 27200390 PMC4852038

[10] Saltychev, M. et al. Pelvic incidence and hip disorders. *Acta Orthop.* **89**, 66–70 (2018). 28914101 10.1080/17453674.2017.1377017 PMC5810835

[11] Gardner, A. et al. The resting coronal and sagittal stance position of the torso in adolescents with and without spinal deformity. *Sci. Rep.* **11**, 2354 (2021). 33504872 10.1038/s41598-021-81818-z PMC7840667

[12] KELLGREN, J. H. & LAWRENCE, J. S. Radiological assessment of osteo-arthrosis. *Ann. Rheum. Dis.* **16**, 494–502 (1957). 13498604 10.1136/ard.16.4.494 PMC1006995

[13] Haffer, H. et al. The Impact of Spinopelvic Mobility on Arthroplasty: Implications for Hip and Spine Surgeons. *J Clin Med*;9. Epub ahead of print August 2020. 10.3390/jcm9082569 10.3390/jcm9082569 PMC7464017 32784374

[14] Moharrami, A. et al. Slight pelvic obliquity is normal in a healthy population: a cross-sectional study. *J. Exp. Orthop.* **10**, 57 (2023). 37254005 10.1186/s40634-023-00613-z PMC10229507

[15] Vignon, E. [Results of the ECHODIAH clinical trial on hip arthrosis]. *Presse Med.* **31**, 7–9 (2002). 11826591

[16] Conrozier, T., Tron, A. M. & Vignon, E. [Coxarthroses]. *Rev. Prat*. **46**, 2201–2205 (1996). 8978176

[17] Conrozier, T. et al. Quantitative measurement of joint space narrowing progression in hip osteoarthritis: a longitudinal retrospective study of patients treated by total hip arthroplasty. *Br. J. Rheumatol.* **37**, 961–968 (1998). 9783760 10.1093/rheumatology/37.9.961

